# The profile of adolescents assisted by the emergency department of a Brazilian private tertiary hospital

**DOI:** 10.1016/j.clinsp.2024.100502

**Published:** 2024-09-19

**Authors:** Alberto Carame Helito, Ricardo Luiz Affonso Fonseca, Ana Helena D'Arcadia de Siqueira, Carol Machado Ferrer, Guilherme Ramos de Faria, Isabella Rocha Morais, Julio Cesar Arnoni Junior, Mateus de Paiva Breziniscki, Christian Valle Morinaga

**Affiliations:** a Emergency Department, Sírio-Libanês Hospital, São Paulo, SP, Brazil; b Resident Physician of the Pediatrics Medical Residency Program at Sírio-Libanês Hospital, São Paulo, SP, Brazil

**Keywords:** Adolescence, Emergency department, Profile, Tertiary hospital, Teenagers, Emergency room, Brazil

## Abstract

**Introduction:**

Adolescents represent an important demographic percentage in the studied population and in Emergency Departments (ED). It is imperative that health professionals and services are prepared to address this population. This work aims to characterize adolescents at the ED of a Brazilian private tertiary hospital.

**Methods:**

The study was an observational, retrospective longitudinal cohort that included 37,450 visits of patients aged 10 to 21 years of age, between January 2018 and June 2022 in the ED of a private tertiary hospital. The study evaluated the reason for the consultation, diagnosis, need for hospitalization, the medical professional responsible for the care, severity, and paying source of care.

**Results:**

53.7% were female. Mean age was 16.2y for girls and 15.6y for boys (p < 0.005). The most common complaints were flu-like symptoms (17.4%), sore throat (8.2%), fever (6.7%) and limb trauma (6.3%). Flu-like symptoms were the main consultations caused in all age groups and genders. 36.8% were attended by a general practitioner, 35.8% by a pediatrician, 15.1% by orthopedics and 5.6% by surgeons. The hospitalization rate was 5.5%. There was a strong correlation between age and hospitalization rate (correlation coefficient [*r* = 0.93]; p < 0.001). The most prevalent diagnoses in hospitalizations were acute abdomen (12.7%) and trauma (9.4%). 78.2% of the consultations were classified as “not urgent”. There was a strong correlation between age and severity (*r* = 0.86; p < 0.001). 92.7% of the consultations were paid by medical insurance.

**Conclusion:**

In this study, flu-like symptoms were the single main reason for adolescents to search for immediate health care, in every age subgroup and gender, but represented a small risk for hospital admission. Limb trauma was more common in younger male teenagers. Acute abdominal pain and trauma were the most frequent causes of hospital admissions. There was a significant and strong correlation between age and both admission rate and severity.

## Highlights

• Adolescent particularities are not always considered for consultations at the ED.
• Flu-like symptoms were the single main reason for adolescents to search for immediate health care.
• Limb trauma was more common in younger and male teenagers.
• Acute abdominal pain/trauma were the most frequent causes of hospital admissions.
• There was a strong correlation between age and both admission rate and severity.

## Introduction

Adolescence comprises the transition period between childhood and adult life, marked by intense changes in physical, mental, emotional, and sexual development.^1^ There are several different definitions described in the literature, which makes research and data collection on this population heterogeneous. The American Academy of Pediatrics (AAP) describes it as the period between ages 11 and 21.^2^ Brain maturation in adolescents usually begins at 10 years of age and can last up to 24 years of age,^3^ a period in which numerous neuronal connections are responsible for socio-emotional regulation and complex cognitive processes, decision-making, sense of well-being and determination of social behaviors and bond with peers.^4^

The field of pediatric emergency emerged from notions of the differences that exist between emergency care for children and adults. Adolescence, located between these two age groups, is little studied in the context of urgencies and emergencies, despite its demographic importance. According to the American Heart Association, the presence of secondary sexual characteristics, specifically breast development in girls and the presence of axillary hair in boys determine the way they should be accessed. From this moment on, patients should be resuscitated following the Advanced Cardiovascular Life Support (ACLS) flowchart, which is the reference for adults.^5^

The authors aim to critically evaluate the information regarding the search for emergency services by this population, focusing on the causes and the profile of patients. The authors expect that some variables will behave differently in each studied age group, as adolescence characterizes the transition from childhood to adulthood.

## Materials and methods

This study was an observational, retrospective, longitudinal cohort and followed the “Strengthening the reporting of observational studies in epidemiology” (STROBE) Statement. The convenience sample included all 37,450 visits of patients between 10 and 21 years of age that took place between January 2018 and June 2022 in the Emergency Department (ED) of a private tertiary hospital, in the city of São Paulo. The information was obtained from a collection of data from medical records in the institution's computerized systems. Patients of other age groups, patients treated in other periods and those who dropped out of care were excluded from the study.

To characterize the sample, the study evaluated the variables: gender, age, day of the week and season, complaint at triage, specialist responsible for the care, diagnosis at discharge or hospitalization, need for hospitalization, classification of severity at triage and source of payment for the emergency care. Diagnosis at discharge or hospitalization, expressed by the International Classification of Diseases (ICD-10) assigned by the attending physician aftercare, was grouped into 28 diagnostic categories, as detailed in Appendix 1. Missing values have been declared in tables where relevant.

The severity classification was determined in the ED triage by the nursing team, based on the document “Reception with Risk Assessment and Classification”, published in 2004 by the Brazilian Ministry of Health.^6^ It subdivides the assistance into “not urgent”, “urgent” and “emergency”.

The criteria “source of payment for the emergency care” included private care, health insurance, and institutional assistance, a health care program aimed at an institution's employees and their dependents.

For the statistical analysis, the association of age with two quantitative variables was performed with Student's *t*-test. Multiple variables were tested using ANOVA or Welch's ANOVA depending on the homogeneity of variance checked by Levene's test.

Correlations between ordinal categorical variables were performed using the Chi-Square Mantel-Haenszel test. Continuous variables were compared using Spearman's correlation test.

Microsoft Excel software for Windows, Version 2016, and R software version 4.0.1 (Vienna, Austria) were used. The statistical significance level was set at p < 0.05.

The present study was approved by the Ethics Committee for Research on Human Beings of the institution (CAAE: 66481823.8.0000.5461), with a waiver of informed consent, as the project has a retrospective design, involving only the collection of the institution's medical records. The researchers preserved the confidentiality of the data.

## Results

The studied sample, containing 37,450 consultations with patients aged 10 to 21 years old, collected between January 2018 and June 2022, represents 9.8% of the 380,531 consultations performed in the ED in the same period. Considering all the appointments included in the study, 53.7% were female. The mean age of girls was 16.2 years (Standard Deviation [SD = 3.54]) and that of boys was 15.6 years (SD = 3.59; p < 0.005). When subdivided by age group, male patients were the majority between 10 and 12 years of age (52.3%). From the age of 13 onwards, the female gender became predominant, with 51.6% between 13 and 15 years of age, 55.4% between 16 and 18 years, and 58.2% between 19 and 21 years of age, as represented in Table 1.

**Table 1:** Gender, total and by age group.

Gender	10‒21 years (n = 37447)	SE	10‒12 years (n = 8545)	SE	13‒15 years (n = 8031)	SE	16‒18 years (n = 8967)	SE	19-21 years (n = 11904)	SE
Female	53.7	0.26	47.7	0.54	51.6	0.56	55.4	0.52	58.2	0.45
Male	46.3	0.26	52.3	0.54	48.4	0.56	44.6	0.52	41.8	0.45

SE, Standard Error (%).

Regarding the complaints presented in the ED triage, Table 2 summarizes the 10 most frequent causes for visiting the ED, in the total sample and by gender and age group. The most common complaints in the service were flu symptoms (17.4%), sore throat (8.2%), fever (6.7%) and limb trauma (6.3%). When separated by gender, some similarities and differences in the epidemiological profile of these populations were well characterized. Flu-like symptoms were the most prevalent cause of consultations in all studied age groups and in both genders. Fever, abdominal pain, limb pain, sore throat, cough for less than 3 weeks, and malaise also appear as relevant causes in all studied groups. Painful urination is one of the top 10 complaints at screening in girls between 16 and 21 years of age, but not in younger girls or adolescent boys. As for intestinal symptoms other than abdominal pain, vomiting is the most frequent complaint in younger adolescents (up to 15 years of age), while diarrhea only shows up as one of the 10 main causes in groups aged 16 years and over, in both genders. Another interesting finding concerns limb trauma. They are the second cause of demand for the ED in boys between 10 and 15 years of age. In boys between 16 and 21 years of age, this complaint remains relevant, but with a lower prevalence. In females, limb trauma appears in the lists of the 10 main reasons for coming to the ED between 10 and 15 years of age, but with lower rates than the male groups. It ceases to be an important cause in girls after the age of 15.

**Table 2:** Main complaints in the Emergency Care triage, total and by age groups (%).

**Both genders**														
**10‒21 years (n = 37450)**		**SE**	**10‒12 years (n = 8545)**		**SE**	**13‒15 years (n = 8031)**		**SE**	**16‒18 years (n = 8967)**		**SE**	**19-21 years (n = 11907)**		**SE**

Flu-like symptoms	17.4	0.20	Flu-like symptoms	13.8	0.37	Flu-like symptoms	16.3	0.41	Flu-like symptoms	19.8	0.42	Flu-like symptoms	19.1	0.36
Sore throat	8.2	0.14	Limb trauma	10.5	0.33	Limb trauma	8.2	0.31	Sore throat	10.6	0.33	Sore throat	8.8	0.26
Fever	6.7	0.13	Fever	10.1	0.33	Fever	8.0	0.30	Fever	5.4	0.24	Malaise	4.7	0.19
Limb trauma	6.3	0.13	Abdominal pain	6.4	0.27	Sore throat	7.7	0.30	Malaise	5.2	0.24	Cough for less than 3 weeks	4.6	0.19
Cough for less than 3 weeks	5.1	0.11	Limb pain	6.4	0.27	Abdominal pain	5.5	0.25	Cough for less than 3 weeks	5.0	0.23	Fever	4.4	0.19
Malaise	4.9	0.11	Cough for less than 3 weeks	5.9	0.25	Malaise	5.5	0.25	Limb trauma	4.3	0.22	Diarrhea	4.3	0.19
Limb pain	4.7	0.11	Sore throat	5.2	0.24	Limb pain	5.2	0.25	Limb pain	4.0	0.21	Dysuria	3.9	0.18
Abdominal pain	4.6	0.11	Malaise	4.3	0.22	Cough for less than 3 weeks	5.1	0.25	Abdominal pain	3.6	0.20	Limb pain	3.8	0.17
Diarrhea	3.2	0.09	Vomiting	3.6	0.20	Headache	3.4	0.20	Diarrhea	3.4	0.19	Abdominal pain	3.4	0.17
Headache	3.1	0.09	Headache	3.3	0.19	Vomiting	3.3	0.20	Headache	3.1	0.18	Limb trauma	3.4	0.17

TOTAL	64.2		TOTAL	69.4		TOTAL	68.2		TOTAL	64.4		TOTAL	60.4	

**Female gender**														

**10‒21 years (n = 20124)**		**SE**	**10‒12 years (n = 4079)**		**SE**	**13‒15 years (n = 4143)**		**SE**	**16‒18 years (n = 4970)**		**SE**	**19-21 years (n = 6932)**		**SE**

Flu-like symptoms	17.4	0.27	Flu-like symptoms	14.3	0.55	Flu-like symptoms	16.8	0.58	Flu-like symptoms	20.3	0.57	Flu-like symptoms	17.6	0.46
Sore throat	9.1	0.20	Fever	9.9	0.47	Sore throat	9.4	0.45	Sore throat	10.9	0.44	Sore throat	9.2	0.35
Fever	5.5	0.16	Limb trauma	8.9	0.45	Fever	6.6	0.38	Malaise	5.9	0.34	Dysuria	6.0	0.29
Malaise	5.2	0.16	Abdominal pain	6.6	0.39	Abdominal pain	6.5	0.38	Cough for less than 3 weeks	5.1	0.31	Malaise	4.5	0.25
Cough for less than 3 weeks	5.0	0.15	Limb pain	6.5	0.39	Limb trauma	6.1	0.37	Abdominal pain	4.0	0.28	Cough for less than 3 weeks	4.5	0.25
Abdominal pain	5.0	0.15	Sore throat	6.1	0.37	Malaise	5.6	0.36	Fever	4.0	0.28	Diarrhea	3.9	0.23
Limb trauma	4.7	0.15	Cough for less than 3 weeks	5.7	0.36	Cough for less than 3 weeks	5.0	0.34	Headache	3.7	0.27	Abdominal pain	3.8	0.23
Limb pain	4.1	0.14	Malaise	4.9	0.34	Limb pain	4.2	0.31	Dysuria	3.7	0.27	Headache	3.5	0.22
Dysuria	3.7	0.13	Vomiting	3.4	0.28	Headache	4.1	0.31	Diarrhea	3.3	0.25	Fever	3.5	0.22
Headache	3.6	0.13	Headache	3.2	0.28	Vomiting	3.3	0.28	Limb pain	3.2	0.25	Limb pain	3.3	0.21

TOTAL	63.2		TOTAL	69.5		TOTAL	67.5		TOTAL	64.2		TOTAL	59.9	

**Male gender**														

**10‒21 years (n = 17323)**		**SE**	**10‒12 years (n = 4466)**		**SE**	**13‒15 years (n = 3888)**		**SE**	**16‒18 years (n = 3997)**		**SE**	**19-21 years (n = 4972)**		**SE**

Flu-like symptoms	17.5	0.29	Flu-like symptoms	13.3	0.51	Flu-like symptoms	15.9	0.59	Flu-like symptoms	19.2	0.62	Flu-like symptoms	21.3	0.58
Limb trauma	8.2	0.21	Limb trauma	12.0	0.49	Limb trauma	10.4	0.49	Sore throat	10.2	0.48	Sore throat	8.3	0.39
Fever	8.1	0.21	Fever	10.4	0.46	Fever	9.5	0.47	Fever	7.1	0.41	Fever	5.6	0.33
Sore throat	7.2	0.20	Abdominal pain	6.3	0.36	Limb pain	6.3	0.39	Limb trauma	6.3	0.38	Malaise	4.9	0.31
Limb pain	5.5	0.17	Limb pain	6.3	0.36	Sore throat	5.9	0.38	Limb pain	5.1	0.35	Diarrhea	4.8	0.30
Cough for less than 3 weeks	5.2	0.17	Cough for less than 3 weeks	6.0	0.36	Malaise	5.4	0.36	Cough for less than 3 weeks	4.9	0.34	Cough for less than 3 weeks	4.7	0.30
Malaise	4.6	0.16	Sore throat	4.4	0.31	Cough for less than 3 weeks	5.3	0.36	Malaise	4.4	0.32	Limb trauma	4.6	0.30
Abdominal pain	4.2	0.15	Vomiting	3.8	0.29	Abdominal pain	4.3	0.33	Diarrhea	3.6	0.30	Limb pain	4.4	0.29
Diarrhea	3.3	0.14	Malaise	3.7	0.28	Vomiting	3.3	0.29	Abdominal pain	3.1	0.27	Abdominal pain	3.0	0.24
Vomiting	2.8	0.13	Headache	3.3	0.27	Headache	2.7	0.26	Headache	2.2	0.23	Vomiting	2.2	0.21
TOTAL	66.6		TOTAL	69.4		TOTAL	69.0		TOTAL	66.0		TOTAL	63.8	

SE, Standard Error (%).

Table 3 summarizes the distribution of consultations concerning the days of the week. For the population the authors studied, Monday was the busiest day at the Emergency Department (16.8% of all visits), while Saturday was the less busy day (11.9%). The day-by-day analysis shows a strong negative correlation between the days of the week and the number of attendances (correlation coefficient [*r* = -0.89]; p < 0.001]. It is noteworthy that the number of visits did rise between Saturday and Monday. As for the season of the year in which the visits happened, 27.6% occurred between March and May (autumn in Brazil), 27.2% between December and February (summer), 23.4% between September and November (spring), and 21.8% between June and August (winter), as represented in Table 4.

**Table 3:** Distribution of consultations by days of the week (Correlation -0.89).

Day of the week	n	%	SE
Monday	6302	16.8	0.19
Tuesday	5936	15.9	0.19
Wednesday	5669	15.1	0.19
Thursday	5463	14.6	0.18
Friday	4640	12.4	0.17
Saturday	4454	11.9	0.17
Sunday	4986	13.3	0.18
TOTAL	37450	100	

SE, Standard Error (%).

**Table 4:** Total number of patients (n) and frequency of Upper airway infection and Trauma diagnosis (%) by season.

	Total			Upper airway infection			Trauma		
Season	n	%	SE	n	%	SE	n	%	SE
Dec-Feb	10180	27.2	0.23	1684	16.5	0.47	1107	10.9	0.45
Mar-May	10333	27.6	0.23	1484	17.4	0.48	1376	13.3	0.49
Jun-Aug	8182	21.8	0.21	1801	18.1	0.49	1017	12.4	0.48
Sep-Nov	8755	23.4	0.22	1335	15.3	0.45	1287	14.7	0.51

SE, Standard Error (%).

Of the total sample, 36.8% were attended by a general practitioner and 35.8% by a pediatrician. Orthopedics was responsible for 15.1% of consultations, while surgeons attended to 5.6% of the studied adolescents (Table 5).

**Table 5:** Medical specialist responsible for care, total and by age groups (%).

10‒21 years (n = 37450)		SE	10‒12 years (n = 8545)		SE	13‒15 years (n = 8031)		SE	16‒18 years (n = 8967)		SE	19-21 years (n = 11907)		SE
GP	36.8	0.25	Pediatrician	72.1	0.49	Pediatrician	71.6	0.50	GP	55.7	0.52	GP	70.1	0.42
Pediatrician	35.8	0.25	Orthopedics	20.5	0.44	Orthopedics	17.7	0.43	Pediatrician	16.4	0.39	Orthopedics	11.4	0.29
Orthopedics	15.1	0.18	Surgery	2.8	0.18	GP	3.5	0.20	Orthopedics	12.4	0.35	Surgery	8.4	0.25
Surgery	5.6	0.12	GP	1.9	0.15	Surgery	2.9	0.19	Surgery	7.1	0.27	Neurology	3.0	0.16
Neurology	1.7	0.07	Neurology	0.0	0.02	Neurology	0.19	0.05	Neurology	2.7	0.17	Cardiology	0.3	0.05
Cardiology	0.1	0.02	Cardiology	0.0	0.00	Cardiology	0.00	0.00	Cardiology	0.23	0.05	Pediatrician	0.3	0.05

TOTAL	95.1		TOTAL	97.3		TOTAL	95.9		TOTAL	94.6		TOTAL	93.5	

SE, Standard Error (%); GP. General Practitioner.

**Fig. 1:**
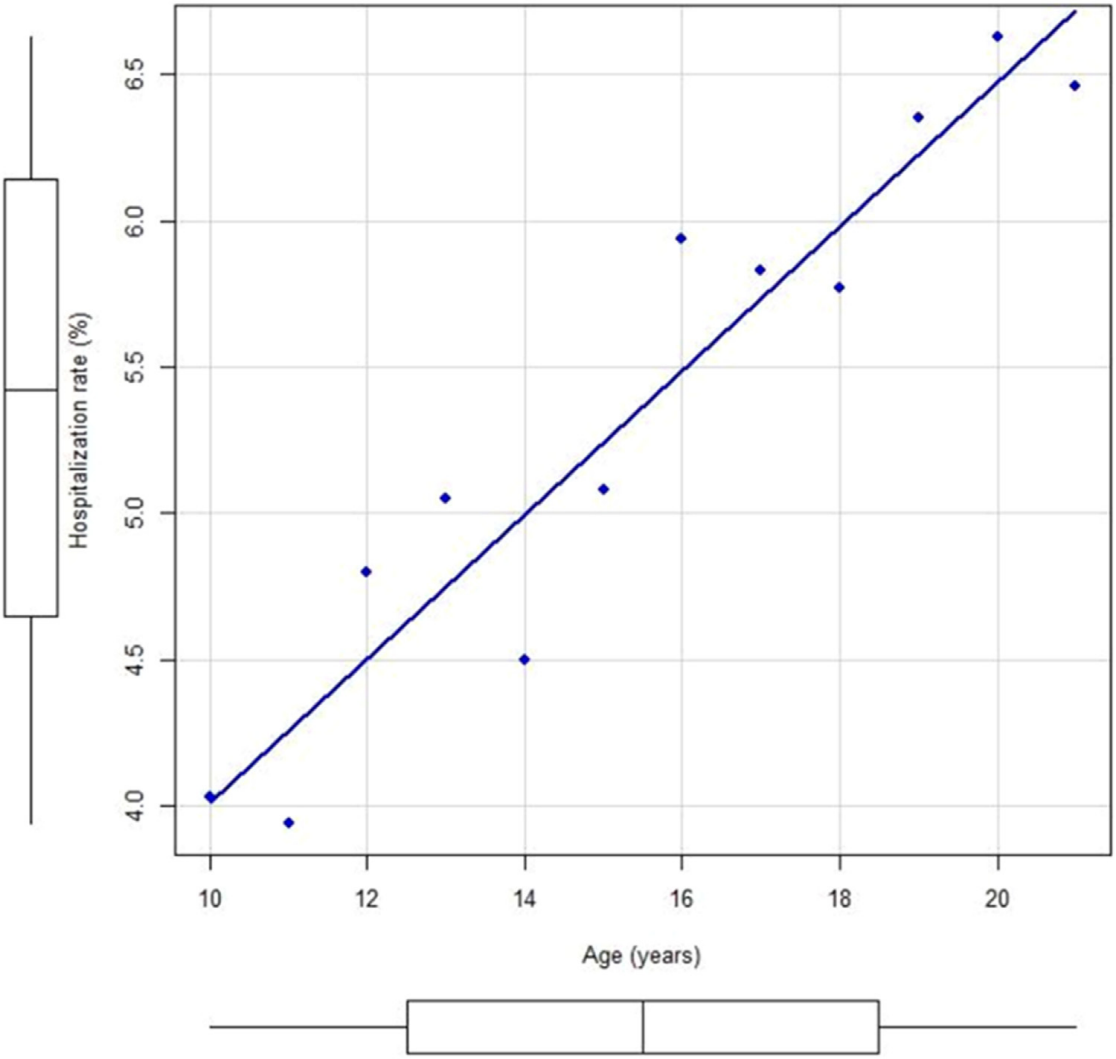
Hospitalization rate and age correlation.

The hospitalization rate of the studied sample was 5.5%. Children from 0 to 9 years of age had a rate of 4.0% in the same period, while adults over 21 years of age were hospitalized in 17.4% of the cases. The overall conversion rate for the period, including all age groups, was 14.1%. The year-by-year analysis of the studied sample shows a strong positive correlation between age and hospitalization rate (correlation coefficient [*r* = 0.93]; p < 0.001), as shown in Fig. 1.

Based on categories of discharge or hospitalization diagnosis, defined by the International Classification of Diseases (ICD-10) assigned by the attending physician aftercare, the most prevalent diagnosis leading to hospitalization was acute abdomen (12.7%), followed by trauma (9.4%) and neurological and psychiatric diseases (8.3%). Table 6 presents the 10 main causes of hospitalization of adolescents received by the ED during the study period. COVID-19 was the diagnosis of 2.1% of hospitalized cases.

**Table 6:** Distribution of hospitalization by diagnosis (%, n = 2050).

Diagnosis	Hospitalization	SE
Acute abdomen	12.7	0.74
Trauma	9.4	0.64
Neurological and psychiatric diseases	8.3	0.61
Renal and urological disease	6.5	0.54
Acute gastroenterocolitis	6.3	0.54
Infectious disease	5.7	0.51
Skin disease	3.7	0.42
Pharyngitis/stomatitis	3.5	0.41
Pulmonary disease	3.1	0.38
COVID-19	2.1	0.32
No ICD	24.0	0.94
TOTAL	85.4	

SE, Standard Error (%).

Table 7 presents, among the 28 studied diagnostic categories, the most prevalent diagnoses at discharge or hospitalization, with their respective hospitalization rates. The authors highlighted the 5 main diagnoses, all corresponding to at least 5% of the total number of visits. They were: upper airway infections (16.8%), trauma (12.8%), pharyngitis/stomatitis (8.5%), acute gastroenterocolitis (8.1%), and COVID-19 (6.4%). The authors also highlighted the 8 diagnoses with hospitalization rates greater than 10%. They were: rheumatologic disease (61.3%), hematologic disease (60.4%), metabolic disease (44.0%), exogenous intoxication (22.8%), liver and bile duct disease (21.1%), acute abdomen (17.4%), heart disease (13.6%) and renal/urological disease (10.3%). Besides acute abdomen (4.0% of consultations) and renal/urological disease (3.5%), none of the diagnoses with a hospitalization rate greater than 10% represented more than 0.4% of the studied visits.

**Table 7:** Main diagnosis and percentage of hospitalization (%, n = 37450).

Diagnosis	% of consultations	SE	% of hospitalization	SE
Upper airway infection	16.8	0.19	0.30	0.07
Trauma	12.8	0.17	4.00	0.28
Pharyngitis/stomatitis	8.5	0.14	2.30	0.27
Acute gastroenterocolitis	8.1	0.14	4.30	0.37
COVID-19	6.4	0.13	1.80	0.27
Acute abdomen	4.0	0.10	17.40	1.0
Renal/urological disease	3.5	0.09	10.30	0.84
Exogenous intoxication	0.34	0.03	22.80	3.7
Heart disease	0.32	0.03	13.60	3.2
Hematological disease	0.14	0.02	60.40	6.7
Rheumatologic disease	0.08	0.01	61.30	8.7
Metabolic disease	0.07	0.01	44.00	9.9
Liver and bile duct disease	0.05	0.01	21.10	9.4

SE, Standard Error (%).

The analysis of the distribution of the two most common diagnoses at discharge/hospitalization by the seasons of the year (Table 4) showed that upper airway infections surpassed trauma in every season. The extent of the difference, though, varied by season. It was higher in the winter (18.1% vs. 12.4%) and lower in the spring (15.3% vs. 14.7%), with statistical significance (p < 0.05).

Regarding the classification of severity level, 78.2% of the consultations were classified as “not urgent”, 11.0% as “urgent” and only 0.75% as “emergency”. There was, however, a strong positive correlation between age and severity level (*r* = 0.86; p < 0.001). While the rate of cases classified as “emergency” was 0.22% in adolescents aged 10 to 12 years, it was up to 5 times higher in individuals aged 19 to 21 years. In the assessment of the risk of hospitalization based on the level of severity determined by the triage of the ED, cases classified as “emergency” were hospitalized in 27.8% of the cases, while “urgent” and “not urgent” had admission rates of 14.2% and 3.8%, respectively.

Table 8 lists the 5 most common discharge/hospitalization diagnoses for each severity level category. The sample representing the “not urgent” level followed the overall pattern of the entire sample, with upper airway infection as the main diagnosis (17.8%), followed by trauma (12.4%), pharyngitis/stomatitis (8.6%), acute gastroenterocolitis (7.7%) and COVID-19 (7.1%). Trauma was the most frequent diagnosis in the group classified as “urgent”. Interestingly, the list of frequent diagnoses for patients whose risk category was “emergency” is almost entirely different. Neurological and psychiatric disease, including seizures, headaches, and central nervous system infections, corresponded to 23.1% of the diagnoses, followed by exogenous intoxication (14.6%), trauma (11.7%), chest pain (10.3%) and heart disease (5.7%).

**Table 8:** Frequency of diagnosis by severity category.

Not urgent	n	%	SE	Urgent	n	%	SE	Emergency	n	%	SE
Upper airway infection	5888	17.8	0.21	Trauma	653	15.8	0.57	Neurological and psychiatric disease	65	23.1	0.65
Trauma	4101	12.4	0.18	Acute gastroenterocolitis	462	11.1	0.49	Exogenous intoxication	41	14.6	0.55
Pharyngitis/Stomatitis	2850	8.6	0.15	Neurological and psychiatric disease	440	10.6	0.48	Trauma	33	11.7	0.50
Acute gastroenterocolitis	2554	7.7	0.15	Upper airway infection	410	9.9	0.46	Chest pain	29	10.3	0.47
COVID-19	2338	7.1	0.14	Pharyngitis/Stomatitis	338	8.2	0.43	Heart disease	16	5.7	0.36

SE, Standard Error (%).

Considering all age groups included in the study, 92.7% of the consultations were paid by medical insurance. Private care corresponded to 5.8% of the total, while institutional assistance corresponded to 1.3%. The mean age of patients whose care was paid for by medical insurance was 15.9 years (SD = 3.57). For patients who had private consultations, the mean age was 16.7 years (SD = 3.52). Institutional consultations had a mean age of 18.3 years (SD = 3.25). There was a significant difference between the mean age values in the different paying sources, with p < 0.001.

## Discussion

São Paulo is appointed as the city with the highest population concentration in Brazil and one of the highest Human Development Indexes (HDI) in the country. The census carried out in 2022 showed that individuals between 10 and 19 years old represented 12.2% of the total population of São Paulo city,^7^ similar to the rest of the state (12.6%) but proportionally lower than the rest of the country (13.8%). Although the data collection for the present study was carried out in the city of São Paulo, only 9.8% of the total attendances corresponded to adolescent patients. This may be a consequence of the socioeconomic bias interfering with the demographic characteristics of the sample, taking into account the profile of patients treated at the studied private institution.

The misuse of primary health services reduces the incentive for healthy life habits and the prevention of risky behaviors, which can increase the chance of acute injuries and feedback on the search for EDs by adolescents. In the United Kingdom, even with access to free-of-charge primary care, adolescents are the lowest users of outpatient services (representing only 5.8% of consultations in primary care) and, disproportionately, are the largest users of EDs (13.9% of consultations in EDs are performed with patients between 10 and 19 years old).^8^ American studies show that the adolescent population prefers to use EDs rather than consult with general practitioners or family and community doctors.^9^ Holland assessed almost 50,000 American adolescents and identified a 24% higher risk of seeking emergency care for those who had not had any routine consultations in the last year, with older adolescents, girls, and those with chronic diseases being the most frequently affected by this situation.^10^ Accordingly, this study also identified a greater demand for emergency care by patients between 16 and 21 years of age (55.7%) and female patients (53.7%).

The greatest demand for care found in the present study, in every age group, was related to flu-like symptoms (fever, runny nose, and cough), corresponding to 17.4% of all patients seen in this unit. Those symptoms may originate from different respiratory infections, such as SARS-CoV-2 and other Coronaviruses, Respiratory Syncytial Virus, Influenza A and B, Parainfluenza, Adenovirus, Rhinovirus, Metapneumovirus and others.^11^^,^^12^ After the consultation, the most common diagnosis assigned by the medical professional was “Upper Airway Infection” (UAI) in every season, representing 16.8% of the ER consultations included in this study. This data contrasts with international literature from the past and the present: North American data from the year 1994 documented the frequency of diagnosed UAI as 1.5% for females and 1.2% for male adolescents. One Irish analysis carried out in 2017 showed that UAIs were the cause of only 1.7% of the visits.^13^ A study from an Italian tertiary-level child's hospital presented UAI as the main diagnosis in 8.3% of cases.^14^ Such remarkable variation of UAI frequencies might be related to social and cultural differences of the studied populations. For some groups, given the self-limited nature of most common colds and mild flu-like symptoms, treatment at home may be preferable. Some families tend to avoid going to ER unless a severe disease is suspected, and might have better access to primary health care or office-based services. Economic reasons might also apply it is noteworthy that 92.7% of the sample was funded by medical insurance plans, which do not always cover the costs of healthcare outside the emergency context.

Although trauma is a prevalent complaint in both genders, the proportion is higher for boys, especially among the younger ones. According to data obtained in the 2019 PeNSE report, the proportion of physically active teenagers in Brazil was higher in males (38.5% vs. 18.0% in girls), which leads to a higher risk of accidents. Boys, for being more exposed to risky behaviors, are also more likely to get involved in other dangerous situations, such as violence. The risk of getting involved in fights with a bladed weapon is twice as high for boys. Examples of other risk situations for trauma are not wearing a seat belt while using vehicles, driving a motor vehicle, and consuming psychoactive substances.^15^ Mortality rates after trauma were reportedly 3.2% in individuals between 10 and 15 years of age and 4.4% in individuals aged 16 and 24 years.^16^ As stated above, an important study carried out in the 1990s in the United States already pointed to trauma as the main diagnosis in ED visits by adolescents.^17^ More recent studies, performed in different countries, confirm this finding and reinforce the global historical importance of trauma as an acute morbidity in adolescents.^13^^,^^14^^,^^18^, ^19^, ^20^ In the present sample, trauma was the second most frequent diagnosis (12.8% of cases), with a risk of hospitalization of 4.0%. It was also the second most frequent cause of hospitalization, representing 9.4% of admissions. Al-Hajj found similar results for pediatric trauma: their male-to-female ratio was 1.8:1 and 4.1% of the studied sample were admitted at their hospital.^21^

Adolescents are a vulnerable group and more likely to report mental health problems, with a high prevalence of medical consultations due to depression, anxiety disorders, and psychotic episodes.^22^ The World Health Organization points to depression as one of the main causes of illness in adolescents.^23^ Deaths by suicide represent the second leading cause of death in individuals between 15 and 19 years of age.^24^ In a document published by the Brazilian Ministry of Health, among the total cases of self-harm in 2019, 9.8% occurred in individuals under 14 years of age and 23.3% in individuals between 15 and 19 years of age.^25^ Such behavior in this age group may be associated with feelings of sadness and hopelessness, depression, anxiety, low self-esteem, physical and sexual abuse, lack of friends and support from relatives, exposure to violence and discrimination in the school environment and the use of psychoactive substances.^26^, ^27^, ^28^ There is evidence that suggests that individuals from Generation Z (also called “iGeneration”, born after 1995) may be more vulnerable because they have less resilience to deal with frustrations and adversities and difficulties in postponing pleasure.^29^^,^^30^ Another Brazilian study that analyzed 259 patients aged up to 18 years who underwent psychiatric care in the ED showed that the majority were female. Girls had more suicide attempts while boys had more episodes of aggression and psychomotor agitation.^31^ The SARS-CoV-2 pandemic has worsened the mental health landscape in society. Leeb described a 31% increase in ED visits by adolescents between 12 and 17 years old for psychiatric causes in 2020, compared to 2019.^32^ In the data collection of the present study, the frequency of consultations for psychiatric causes was also higher in 2020 than in 2019, but without statistical significance (1.25% vs. 1.04%, p = 0.23), and returned to the baseline value in 2021 (1.01%, p = 0.24). The authors emphasize that the hospital evaluated by the present study is not considered a reference in psychiatric emergencies or mental health hospitalizations. Even so, there were 365 consultations due to psychiatric conditions or exogenous intoxication in adolescents during the studied period. This reinforces the importance of health teams in general hospitals to be trained to deal with psychiatric urgencies and emergencies and include qualified psychologists and psychiatrists.

One limitation of this study was the specific sociodemographic profile of the sample, which may not represent the entire population of São Paulo city. Another limitation is the lack of longitudinal information, as the outcome of patients was not studied. The authors also weren't able to determine which patients had chronic conditions, and what could enlighten risk factors for severity and hospital admission. The categorization of patients was based on the assigned ICD-10, and not on specific diagnostic criteria for each disease. Finally, many of the diagnostic categories were not accessed following well-defined institutional protocols which may lead to different treatment strategies.

## Conclusion

Outlining the characteristics and demands of the adolescent population is essential since their particularities are not always considered at the time of consultation at the ED. In the present study, flu-like symptoms were the single main reason for adolescents to search for immediate health care, in both genders and in every age subgroup, but represented a very small risk for hospital admission. Limb trauma was more common in younger and male teenagers. Acute abdominal pain and trauma were the most frequent causes of hospital admissions. There was a significant and strong correlation between age and both admission rate and severity in the studied sample. These results should help in the delineation of standardized assistance protocols aimed at this very specific population in the ED, which should improve patient experience and clinical outcomes.

## Declaration of competing interest

The authors declare no conflicts of interest.

## Appendix Supplementary materials

Image, application 1

## Footnotes

Supplementary material associated with this article can be found, in the online version, at doi:10.1016/j.clinsp.2024.100502.
