# Supplementary material for: The profile of adolescents assisted by the emergency department of a Brazilian private tertiary hospital
Source: Clinics (Sao Paulo). 2024 Sep 19;79:100502. doi: 10.1016/j.clinsp.2024.100502 (PMC11437818; doi:10.1016/j.clinsp.2024.100502)
Supplement: Supplementary file 1 [file mmc1.docx]

**Appendix 1: Diagnostic Categories**

**ACUTE ABDOMEN**Abdominal distension

Abdominal pain

Acute appendicitis

Acute pancreatitis

Mesenteric lymphadenitis

**ACUTE GASTROENTEROCOLITIS**

Food poisoning

Gastroenterocolitis (infectious and non-infectious)

Intestinal parasitosis

Nausea and vomiting

**CHEST PAIN**

Chest pain (ventilatory and non-ventilatory)

**COVID-19**

Infection by SARS-CoV-2

**EAR AND NOSE DISEASE**Acute and chronic mastoiditis

Acute and chronic serous otitis

Acute myringitis

Acute otitis externa

Acute otitis media

Allergic rhinitis

Barotraumatic otitis

Ear and nose perichondritis

Epistaxis

External ear abscess

Foreign body in the ear or nose

Impacted cerumen

Nasal septum deviation

Otalgia

Otorrhagia

Outer ear cellulitis

Tympanic membrane perforation

Vasomotor rhinitis

**EXOGENOUS INTOXICATION**

Exogenous intoxication by drugs

**GASTROINTESTINAL DISEASE**Constipation

Crohn's disease

Dyspepsia

Dysphagia

Esophageal, gastric and duodenal ulcer

Esophagitis

Fecal incontinence

Functional diarrhea

Gastritis

Gastroesophageal reflux disease

Gastrointestinal bleeding

Hematemesis

Intestinal malabsorption

Irritable bowel syndrome

Melena

Ulcerative colitis

**GYNECOLOGICAL DISEASE**Acute mastitis

Acute vulvovaginitis

Bartholin's gland cyst

Breast cyst

Breast hypertrophy

Complications of intrauterine devices

Dysmenorrhea

Female genital tract fistula

Foreign body in the vulva and vagina

Galactorrhea

Genital candidiasis

Hyperemesis gravidarum

Hypermenorrhagia

Mastalgia

Menstrual irregularity

Ovarian cyst and its complications

Parametritis

Pelvic inflammatory disease

Pregnancy-related conditions

Premenstrual syndrome

Threatened abortion

Topical and ectopic pregnancy

Vaginal bleeding

Vulvar abscess

Vulvar cysts

Vulvar pruritus

Vulvar ulcer

**HEART DISEASE**Acute endocarditis

Acute myocarditis

Acute pericarditis

Bradyarrhythmia

Cardiogenic shock

Systemic arterial hypertension

Tachyarrhythmias

**HEMATOLOGICAL DISEASE**Anemia

Bone marrow aplasia

Coagulopathies

Cytopenias

Embolism

Hyperproliferative anemias

Leukemias

Thrombophilias

Thrombosis

**HYDROELECTROLYTIC DISORDERS**Acid-base balance disorders

Dehydration

Electrolyte disorders

Hypovolemic shock

**INFECTIOUS DISEASE**

Anogenital warts

Arbovirus infection (dengue, zika, chikungunya)

Contact or exposure to infectious diseases

Enterovirus infection

Exanthematous disease (measles, rubella, scarlet fever, roseola infantum, infectious erythema, chickenpox)

Genital ulcer

Infectious arthritis

Lymphadenitis

Mononucleosis

Osteomyelitis

Sepsis

Sexually transmitted infections

Syphilis

Toxic shock syndrome

Unspecified fever

**LIVER AND BILIARY DISEASE**Acute and chronic liver failure

Acute cholecystitis

Cholangitis

Gallstones and bile duct stones

Gilbert's syndrome

Hepatitis

Portal hypertension

**METABOLIC DISEASE**

Calcium metabolism disorders

Diabetes Insipidus

Diabetes Mellitus

Hypo/hyperglycemia

Obesity

Puberty disorder

Thyroid disorders (hyperthyroidism, hypothyroidism, thyroiditis)

**NEUROLOGICAL AND PSYCHIATRIC DISEASE**

Anxiety disorders

Brain cysts and tumors

Brain hemorrhages

Cerebellar disorders

Cranial nerve disorders

Dizziness

Dystonias

Eating disorders (anorexia nervosa and bulimia nervosa)

Encephalitis

Epileptic disorders

Febrile seizures

Headache

Hearing loss

Hydrocephalus

Labyrinthitis

Meningitis

Mood disorders

Movement disorders

Multiple sclerosis

Muscular dystrophy

Myopathies

Neurodevelopmental disorders

Personality disorders

Psychotic disorders

Radiculopathies e neuropathies

Self-aggression

Sleep disorders

Somatoform disorders

Stroke (ischemic or hemorrhagic)

Syncope and collapse

Wernicke’s encephalopathy

**NON-SPECIFIED ALLERGY**

Anaphylaxis

Angioedema

**OPHTHALMOLOGICAL DISEASE**Blepharitis

Chalazion

Conjunctival bleeding

Conjunctivitis

Corneal ulcer

Eye pain

Eyelid disorders

Hordeolum

Keratitis

Ocular foreign body

Visual symptoms (diplopia, loss of vision)

**OSTEOMUSCULAR DISEASE**

Arthritis

Calcaneal spur

Cramps and spasms

Fasciitis

Iliotibial band syndrome

Joint effusion

Joint pain

Ligament disorders

Limb deformities (congenital and acquired)

Medial and lateral epicondylitis

Muscle disorders

Myalgia

Myositis

Osteochondrosis

Osteonecrosis

Palmar and plantar fibromatosis

Patellofemoral disorders

Radiculopathies

Rotator cuff syndrome

Sacrococcygeal disorders

Scoliosis

Spondylolisthesis

Spondylolysis

Synovitis

Temporomandibular joint disorders

Tendinitis

Torticollis

**OTHER PAINS**

Acute pain

Unspecified pain

**PHARYNGITIS/STOMATITIS**

Disease of the adenoids

Dysphagia

Foreign body in the pharynx

Gingivitis

Gingivostomatitis

Glossitis

Mouth ulcer

Peritonsillar abscess

Pharyngitis

Pharyngotonsillitis

**PULMONARY DISEASE**

Acute and chronic bronchitis

Bronchiectasis

Cystic fibrosis

Hemoptysis

Hiccup

Pneumonia

Pneumothorax

Pulmonar embolisms and thrombosis

Respiratory failure

**RENAL AND UROLOGIC DISEASE**

Acute or chronic renal failure

Cystitis

Glomerular syndromes

Hematuria

Hydrocele

Phimosis and paraphimosis

Priapism

Pyelonephritis

Spermatic cord torsion

Urethritis

Urinary incontinence

Urinary lithiasis (kidney, ureter, bladder and urethra)

Urinary retention

**RHEUMATOLOGICAL DISEASE**

Autoimmune disease

Collagenosis

Vasculitis

Osteochondropathies

**SKIN DISEASE**Acne

Atopic dermatitis

Bruises and ecchymoses

Bullous diseases

Cutaneous granuloma

Cutaneous hemangioma

Dyshidrosis

Dermatozoonosis (larva migrans, pediculosis, scabies, myiasis)

Fungal skin infections

Pharmacodermias

Pityriasis rosea

Psoriasis

Pyoderma (folliculitis, impetigo, cellulitis, erysipelas, panniculitis)

Rosacea

Skin abscess

Skin pruritus

Subcutaneous cysts (lipoma, pilonidal cyst)

Urticaria

Viral warts

Xeroderma

**SURGICAL DISEASE**

Abdominal hernias (inguinal, umbilical, femoral)

Anorectal disease

Bowel obstruction

Complication of surgical wounds

Foreign body in the airway

Foreign body in the gastrointestinal tract

Gastrostomy care

Ingrown toenail

Masses and tumors in the gastrointestinal tract

Phlebitis and thrombophlebitis

Surgical wound care

Tracheostomy care

Urethral stricture

**TRAUMA**

Abdominal trauma

Aggression

Bites

Bone dislocation

Burns

Chest trauma

Corrosive injury

Genitourinary tract trauma

Joint strain and sprain

Ligament rupture

Limb fractures

Muscle sprain

Nasal trauma

Oral trauma

Pelvic trauma

Sequelae of fractures

Spinal trauma

Stabbing wounds

Traffic accidents

Trauma to the skull and face

Upper and lower limb trauma

Work accident

**UPPER AIRWAY INFECTION**

Acute nasopharyngitis

Anosmia

Cough

Dysphonia

Influenza virus infection

Laryngitis

Sinusitis

Tracheitis

**OTHERS**

Other diagnoses, not included in the previous categories
